# Improved resolution in single-molecule localization microscopy using QD-PAINT

**DOI:** 10.1038/s12276-021-00572-4

**Published:** 2021-03-02

**Authors:** Yeonho Chang, Do-Hyeon Kim, Kai Zhou, Min Gyu Jeong, Soyeon Park, Yonghoon Kwon, Triet Minh Hong, Jungeun Noh, Sung Ho Ryu

**Affiliations:** 1 Department of Life Sciences, Pohang University of Science and Technology, Pohang, 37673 Republic of Korea; 2 Integrative Biosciences and Biotechnology, Pohang University of Science and Technology, Pohang, 37673 Republic of Korea

**Keywords:** Fluorescence imaging, Quantum dots, Oligonucleotide probes, Fluorescent dyes, Super-resolution microscopy

## Abstract

Single-molecule localization microscopy (SMLM) has allowed the observation of various molecular structures in cells beyond the diffraction limit using organic dyes. In principle, the SMLM resolution depends on the precision of photoswitching fluorophore localization, which is inversely correlated with the square root of the number of photons released from the individual fluorophores. Thus, increasing the photon number by using highly bright fluorophores, such as quantum dots (QDs), can theoretically fundamentally overcome the current resolution limit of SMLM. However, the use of QDs in SMLM has been challenging because QDs have no photoswitching property, which is essential for SMLM, and they exhibit nonspecificity and multivalency, which complicate their use in fluorescence imaging. Here, we present a method to utilize QDs in SMLM to surpass the resolution limit of the current SMLM utilizing organic dyes. We confer monovalency, specificity, and photoswitchability on QDs by steric exclusion via passivation and ligand exchange with ptDNA, PEG, and casein as well as by DNA point accumulation for imaging in nanoscale topography (DNA-PAINT) via automatic thermally driven hybridization between target-bound docking and dye-bound complementary imager strands. QDs are made monovalent and photoswitchable to enable SMLM and show substantially better photophysical properties than Cy3, with higher fluorescence intensity and an improved resolution factor. QD-PAINT displays improved spatial resolution with a narrower full width at half maximum (FWHM) than DNA-PAINT with Cy3. In summary, QD-PAINT shows great promise as a next-generation SMLM method for overcoming the limited resolution of the current SMLM.

## Microscopy: Seeing individual molecules inside cells with improved resolution

A new microscopy method called QD-PAINT will allow researchers to see inside cells with nanoscale precision. The method combines two existing technologies, DNA-PAINT and steric exclusion, to show a proof-of-concept of a proper utilization of quantum dots (QDs) in single-molecule localization microscopy (SMLM) with improved resolution. In the last decade, SMLM using organic dyes allowed observation of previously invisible biological phenomena, such as individual proteins, but resolution was limited to around 20 nm. QDs emit much brighter fluorescence than organic dyes and could theoretically improve the resolution of SMLM. Sung Ho Ryu and Do-Hyeon Kim at Pohang University of Science and Technology in South Korea adapted QDs for SMLM, conferring them with photoswitchability and specificity, and improved SMLM resolution to about 8 nm. The researchers suggest the method could be fine-tuned for even higher resolution.

## Introduction

Single-molecule localization microscopy (SMLM) has become a popular technique to investigate the molecular structures, spatial distribution, clustering, and diffusion of receptors below the diffraction limit of light, allowing the observation of many biological phenomena never seen prior to the realization of these breakthrough techniques^1–4^. SMLM techniques share the common principle that a small subset of fluorophores labeling target proteins are spatiotemporally separated by switching their emission between a fluorescent state (“on”) and a nonfluorescent state (“off”), allowing sequential detection and localization of individual fluorophores^5,6^. In DNA point accumulation for imaging in nanoscale topography (DNA-PAINT), the fluorescence transition is achieved by automatic thermally driven hybridization between the target molecule-attached ‘docking’ strands and the fluorophore-attached complementary ‘imager’ strands^7^.

In principle, the spatial resolution of SMLM depends on the uncertainty of photoswitching fluorophore localization, which is inversely proportional to the square root of the number of detected photons^8–11^. The current SMLM techniques frequently utilize photoswitchable organic dyes that allow achievable resolutions of 20–40 nm at best^12^. The resolution limit is attributed to the fundamental photophysical characteristics of organic dyes with a limited number of photons released during the fluorescence transition between fluorescent and nonfluorescent states^8,13^. In other words, a fluorescent label with a higher photon output can further fundamentally increase the spatial resolution^14^.

Quantum dots (QDs), as one of the brightest fluorophores, are renowned for their potential as next-generation fluorophores in a broad spectrum of biological imaging applications^15–18^. QDs are known to be 10–20 times brighter than organic dyes^19,20^. This brightness of QDs arises because their extinction coefficients are 10–50 times larger than those of organic dyes, allowing the absorption of 10–50 times more photons than organic dyes at the same excitation photon flux^21^. Furthermore, QDs are hundreds to several thousands of times more photostable than organic dyes, indicating that QDs can tolerate much higher excitation photon flux and resist photobleaching^19,21^. In theory, QDs with 10–20 times higher fluorescence intensity than organic dyes can achieve an ~3–5-fold improvement in resolution. Despite these excellent photophysical properties, an SMLM method that can distinguish many different molecules within the diffraction-limited region while taking advantage of the full potential of the substantially high photon yield of QDs to improve the resolution beyond the current limit of SMLM has not been reported^22–24^. QDB3 utilizes the ‘uncontrolled’ intrinsic blinking of QDs for very short periods, limiting the number of QDs that can be distinguished within the diffraction-limited region, thereby making this technique difficult to extend to superresolution imaging. QSTORM induces the stochastic blueing of QDs upon laser illumination at high power in a ‘controlled’ manner by altering the oxygen concentration with 10 or 20% glycerol. In QSTORM, the blueing process decreases the fluorescence intensity of QDs, leading to a 24 nm resolution for individual QDs (FWHM) being obtained on microtubules. Furthermore, QD-utilizing SMLM on proteins in cells has been challenging. First and most importantly, QDs are not photoswitchable, which is essential for SMLM techniques to achieve the necessary stochastic switching on and off of subsets of QDs for sequential detection and localization of each QD within the diffraction limit^23^. Second, QDs are nonspecific and multivalent, which complicates the use of QDs by potentially inducing undesired on- or off-target effects such as oligomerization, activation, internalization, or redistribution of molecules^25^. If QDs with a substantially high photon output could be properly utilized for SMLM by conferring photoswitchability, specificity, and monovalency on them, then the current resolution limit in photoswitchable organic dye-utilizing SMLM would be overcome.

Here, we developed a method to increase the resolution of conventional SMLM utilizing organic dyes by employing the highly bright property of QDs in DNA-PAINT, named QD-PAINT. We conferred monovalency, specificity, and photoswitchability on QDs via steric exclusion and DNA-PAINT. We analyzed the fluorescence intensity of ptDNA-PEG-casein-passivated mQDs, which was superior to that of Cy3. We reconstructed images of single molecules in QD-PAINT that showed a narrower full width at half maximum than that in DNA-PAINT with Cy3.

## Materials and methods

### Reagents

Organic QD585 (#Q21711MP) and Lipofectamine LTX (#15338100) were purchased from Invitrogen. Glass coverslips were purchased from Marienfeld Laboratory Glassware (25 mm, #0111580). Tetrabutylammonium bromide (TBAB, #426288), chloroform (#C2432), fibronectin (#F2006), hydrofluoric acid (#695068), and casein (#C6554) were purchased from Sigma-Aldrich. 2,5,8,11,14,17,20-Heptaoxadocosane-22-thiol (mPEG thiol) was purchased from Polypure (#11156–0695). Carboxy PEG6 alkane thiol, or HS-(CH_2_)_11_-(OCH_2_CH_2_)_6_-OCH_2_CO_2_H (HSC_11_EG_6_CO_2_H), was purchased from ProChimia (#TH003-m11.n6-0.1). Sodium hydroxide was purchased from ACROS (#S/4845). Sephadex NAP5 (#17085301) and NAP10 (#17085401) columns were purchased from GE Healthcare. A 30 kDa Centricon spin column was purchased from Amicon (#Z717185). A Fixation/Permeabilization Solution Kit was purchased from BD Biosciences (#554714). Acetone was purchased from Samchun Chemical (#A0097). Absolute ethanol was purchased from Merck (#1.00983.1011). Bovine serum albumin was purchased from Affymetrix (#9048-46-8). Modified DNA oligonucleotides for docking and imager strands were purchased from Integrated DNA Technologies (Supplementary Table 1). The docking strands (2 mM) with NH2 in HEPES buffer (200 mM, pH 8.5) were reacted with BG-GLA-NHS (20 mM, #S9151S, New England Biolabs) in anhydrous dimethyl sulfoxide (DMSO, #D8418, Sigma-Aldrich) at RT for 30 min according to the manufacturer’s instructions (New England Biolabs).

### Phase transfer of organic QDs into the aqueous phase

Organic QD585 (600 µl in chloroform) was mixed with TBAB (400 µl, 0.3 M in chloroform) in a 5 ml glass vial, and then, mPEG thiol (CH3O(CH2CH2O)6C2H5SH) (36 µl, neat) was slowly added dropwise. After shaking O/N, aqueous NaOH (800 µl, 0.2 M) was added to the mixture and shaken for 30 min. The successfully phase-transferred orange-colored aqueous particles were positioned on top of the denser and clear organic phase. The orange-colored aqueous-phase particles were collected and pre-equilibrated via a Sephadex NAP10 desalting column with elution buffer (10 mM Tris, 30 mM NaCl, pH 8.0). The buffer-exchanged particles were concentrated with a Centricon spin column (30 kDa molecular weight cutoff). The QD concentration was measured in a NanoDrop 2000 based on the absorbance at 350 nm (the extinction coefficient of QD585 is 3,500,000 M^−1^ cm^−1^).

### Preparation of mQDs

For the wrapping of QDs with ptDNA at a 1:1 stoichiometry, 0 (50 µl, 10 mM Tris 30 mM NaCl buffer), 0.5 (50 µl, 100 nM), and 1 (50 µl, 200 nM) equivalents of ptDNA were slowly added dropwise to the phase-transferred QDs (100 µl, 100 nM) under vigorous stirring (for the sequence, see Supplementary Table 1). After shaking O/N, 10 µl of the ptDNA-wrapped QDs was removed and run on an analytical agarose gel (0.8% in sodium borate buffer) at 100 V for 10 min. After the complete conjugation of all QDs with ptDNA, the surface ligands were exchanged with carboxy PEG6 alkane thiol ((CO2H)CH2O(CH2CH2O)6C11H23SH) in 10 mM Tris 30 mM NaCl buffer (pH 8.0) for 10 min. Then, 0.5 ml of the PEG6-ptDNA-QD solution was pre-equilibrated with elution buffer (10 mM Tris, 30 mM NaCl, pH 8.0) by using a Sephadex NAP5 column to remove excess alkane PEG6 thiol. The QDs were concentrated and collected with a Centricon spin column (30 kDa molecular weight cutoff) for storage at 4 °C. Prior to use for imaging, the QDs were incubated with 0.5% casein to further reduce nonspecific binding to cells.

### Plasmid DNA

SNAP-EGFR was prepared as previously described^26^.

### Sample preparation

COS7 cells (American Type Culture Collection, ATCC) were cultured in Dulbecco’s modified Eagle’s medium (DMEM, 12-604F, Lonza) supplemented with 10% (v/v) FBS (Gibco) at 37 °C, 5% CO_2_, and 95% humidity in a 6-well plate. Transient expression of SNAP-EGFR was achieved by plasmid transfection using Lipofectamine LTX according to the manufacturer’s protocol. After 36 h, the cells were treated with 1 μM BG-docking strands for 30 min and then washed three times with PBS. The cells were detached with 1 mM EDTA and then seeded onto a 25 mm glass coverslip in phenol red-free DMEM with 10% FBS. For cell fixation, the phenol red-free DMEM was removed, and the coverslips were rinsed with PBS. Then, the cells were fixed with 4% paraformaldehyde and 0.1% glutaraldehyde in PBS for 15 min at 37 °C and washed three times with PBS. For cell fixation and permeabilization, the coverslips were rinsed with PBS after removal of the phenol red-free DMEM. The cells were fixed and permeabilized with fixation/permeabilization buffer containing 0.1% glutaraldehyde, incubated for 30 min at 37 °C, and washed once with washing buffer. Then, the cells were incubated with washing buffer containing 0.1% NaBH4 for 30 min and washed once with washing buffer and three times with PBS.

### Microscope setup

Fluorescence imaging was carried out on a homemade objective-type total internal reflection fluorescence (TIRF) microscope built on an inverted microscope (IX-81, Olympus) equipped with an XYZ automated stage (MS-2000, Applied Scientific Instrumentation). A 561-nm laser (YLK 6150T, Lasos) was aligned with an oil-immersion TIRF objective lens (APON 100XOTIRF/1.49, Olympus). The fluorescence from QDs and Cy3 was collected by an electron multiplying charge-coupled device (EM-CCD) camera (iXon Ultra 897, Andor Technology) in an adaptor (TuCam, Andor Technology). A 1.6X amplifier and a 1.43X tube lens were used to increase the magnification. All instrument operations and data acquisitions were controlled by MetaMorph (Molecular Devices) and custom plug-ins written in MATLAB (MathWorks).

### Imaging of SNAP-EGFR by using mQDs and Cy3 in DNA-PAINT

Glass coverslips (25 mm) were cleaned by sonication in a water bath (1510R-DTH, Branson) with deionized water for 5 min, acetone for 30 min, and 1% hydrofluoric acid for 15 min. Then, the coverslips were thoroughly rinsed more than 20 times with deionized water to remove all traces of hydrofluoric acid. Next, the coverslips were sterilized in ethanol under UV light for 30 min and washed three times with PBS. The coverslips were coated with fibronectin (100 µg/ml) dissolved in PBS for 1 h prior to seeding COS7 cells expressing SNAP-EGFR labeled with BG-docking strands (for the sequence, see Supplementary Table 1). Prior to treatment with 0.5% casein-passivated mQDs (200 pM–2 nM), the fixed, fixed and permeabilized, or live cells were washed once with 1% BSA in PBS with 300 mM NaCl (pH 8.0) and incubated with 3% BSA in PBS or DMEM with 300 mM NaCl (pH 8.0) for 20 min to reduce the nonspecific binding of mQDs to cells and glass. The cells were incubated with mQDs or Cy3-bearing imager strands for 20 min and TIR illuminated using a 561-nm laser with an excitation intensity of 150 W/cm^2^ (for mQDs) or 10–20 W/cm^2^ (for Cy3) for an exposure time of 500 ms (for Cy3) or 5000 ms (for mQDs).

### On-time of mQDs and Cy3 in SMLM

The number of frames for which single molecules of mQDs or Cy3 were fluorescent, or switched ‘on’, was measured. The on-frame number was multiplied by the exposure time to calculate the on-time of mQDs and Cy3. The on-times of individual fluorescent molecules were fitted by exponential decay curves to obtain the mean on-time.

### Fluorescence intensity profiles of mQDs and Cy3

The single-molecule intensities of mQDs and Cy3 were measured by using MATLAB. The statistical distribution of fluorescence intensity per fluorescence population density during SMLM imaging in QD-PAINT and DNA-PAINT with Cy3 was plotted.

### Reconstruction and measurement of the FWHM

mQD- and Cy3-utilizing single-molecule localization microscopy was performed as previously described^2^. Subsequently, the FWHM of the point spread function of each reconstructed mQD or Cy3 signal was calculated in MATLAB.

## Results

### Specific and monovalent quantum dots enable fluorescence imaging in cells via DNA hybridization

**Fig. 1:**
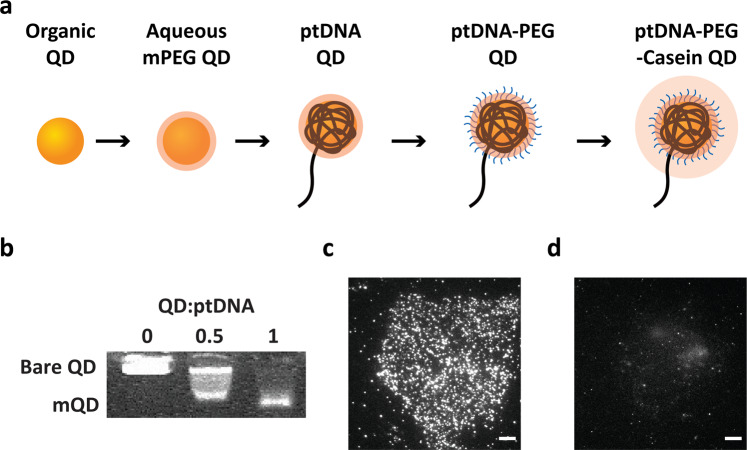
Formation of monovalent QDs for fluorescence imaging in cells. **a** Experimental scheme of the formation of mQDs for imaging in cells. **b** Agarose gel electrophoresis of ptDNA-wrapped QDs yielding monovalent products. Representative TIRF images of COS7 cells expressing SNAP-EGFR with (**c**) and without (**d**) BG-docking strands attached obtained by using mQDs bearing complementary imager strands via 20-nucleotide hybridization. Scale bars, 5 μm.

To produce specific and monovalent QDs (mQDs) by steric exclusion^27^, CdSe:ZnS QDs that emit at 585 nm (QD585) were phase transferred from the organic to aqueous phase and wrapped with a polymer, a 50-adenosine phosphorothioate DNA (ptDNA, A^s^_50_, where ‘S’ refers to sulfur modification on the DNA backbone) domain with a 20-nucleotide linker strand and a 20-nucleotide imager strand (Fig. 1a). In comparison with the unwrapped bare QDs (0:1 ptDNA:QDs) and partially wrapped QDs at a 0.5:1 ratio of ptDNA:QDs, the ptDNA-wrapped mQDs at a 1:1 stoichiometric ratio moved toward the positively charged pole in a single band in gel electrophoresis, indicating the complete conjugation of all QDs with negatively charged ptDNA and the production of a single species (Fig. 1b). The ptDNA-wrapped mQDs were passivated with commercially available polyethylene glycol (carboxy PEG6 alkane thiol) ligands by ligand exchange and then with 0.5% casein immediately before imaging (Fig. 1a). The hydrodynamic diameter of the mQDs (QD585) including the imager strand was estimated to increase from ~5 to 15 nm, as measured and estimated by using dynamic light scattering as in previous reports^27,28^. We next applied imager strand-bearing ptDNA-PEG6-casein-passivated mQDs to imaging of SNAP-EGFR covalently attached to O^6^-benzylguanine (BG)-conjugated 20-nucleotide complementary docking strands at a 1:1 stoichiometry. The small-sized SNAP-tag (~5 nm) allowed a decrease in the linkage error compared to traditional primary and DNA-conjugated secondary antibody labeling (~20 nm, as the size of a typical IgG antibody is ~10 nm). Determination of the spatial organization of EGFR on the plasma membrane is essential for understanding its physiological and pathological roles^29^ and has been investigated by using SMLM techniques such as dSTORM^30^ and Exchange-PAINT^31^. We also chose EGFR as a target molecule to apply QD-PAINT and test its applicability in cell imaging with improved spatial resolution compared with the current SMLM resolution. The large size of the mQDs with long ptDNA-linker imager strands hindered their binding to the central zone of fixed cells (Supplementary Fig. 1). An organic dye, Cy3, bearing a 20-nucleotide linker and a 20-nucleotide imager strand, also showed reduced binding to the central zone of cells at low concentrations (Supplementary Fig. 2). Although Cy3 bearing long imager strands could bind throughout the cells at highly increased concentrations, unfortunately, the mQDs had the fundamental issue that their concentration could not be increased above a certain level due to their high brightness, which would increase the overall background signal and thus affect the imaging quality. Therefore, while keeping the mQD concentration low, we fixed and permeabilized cells, which allowed both mQDs and Cy3 with long imager strands to show improved binding throughout the cells (Fig. 1c and Supplementary Fig. 3). Next, we wanted to ensure that the mQD binding is specific to SNAP-EGFR, not to other proteins or to the glass surface. Because mQDs are immobile upon binding to SNAP-EGFR, other molecules, or the glass surface for fixed-permeabilized cells, we applied mQDs to live SNAP-EGFR-expressing COS7 cells. In live cells, the mQDs were primarily mobile (Supplementary Video 1), ruling out the possibility of mQD nonspecific binding to glass surfaces. In addition, in the absence of the BG-docking strands, the mQDs showed low nonspecific binding, suggesting that the mQD binding to both mobile and immobile proteins on the plasma membrane is specific to SNAP-EGFR via hybridization between the docking and imager strands (Fig. 1d).

### Photoswitchable mQDs in QD-PAINT are superior to Cy3 in DNA-PAINT

**Fig. 2:**
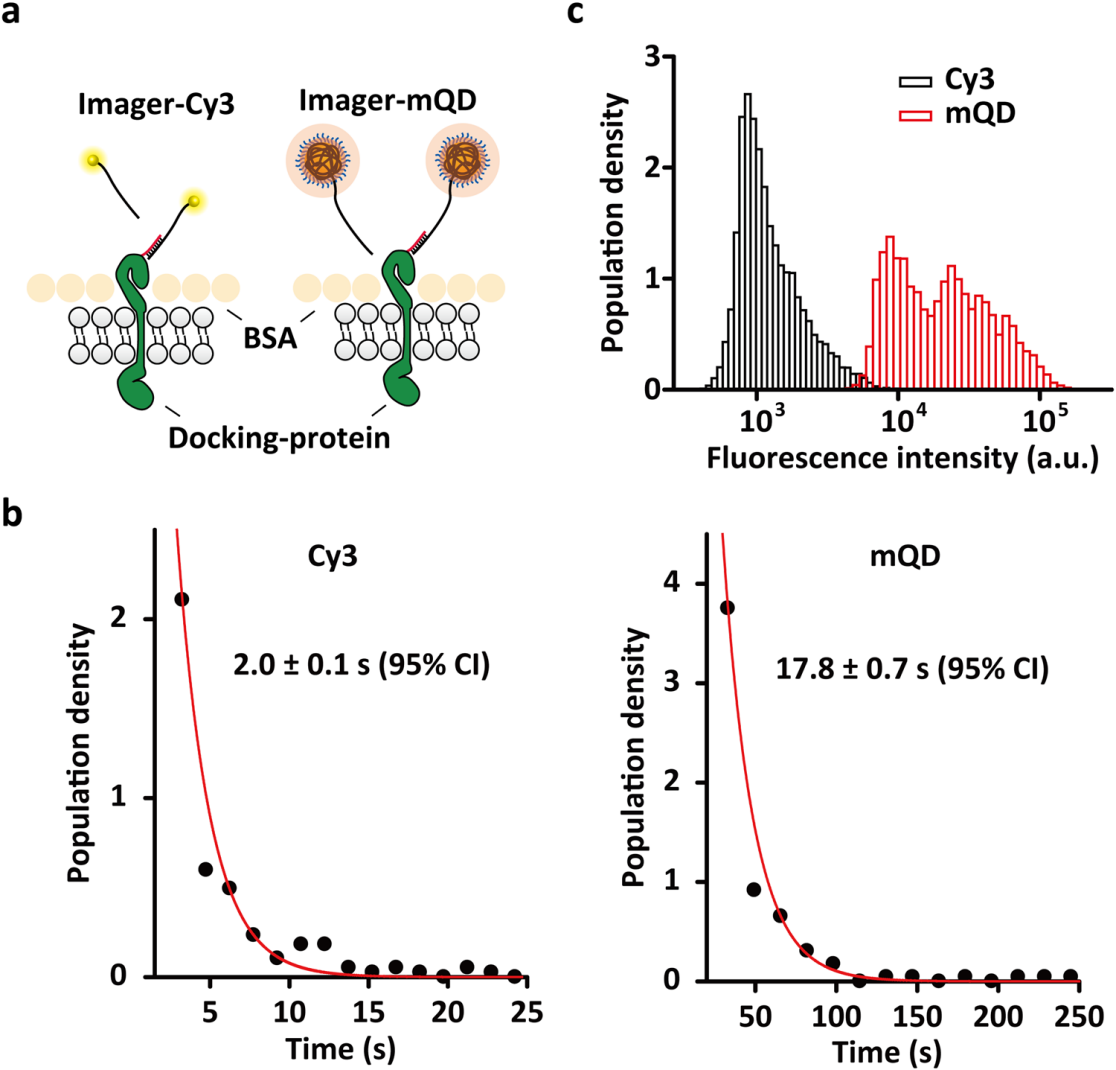
QD-PAINT and DNA-PAINT with Cy3. **a** Schematic of conducting DNA-PAINT with Cy3 (left) and QD-PAINT (right) on cells. **b** Plots of single-molecule measurements of the on-time of mQDs (left) and Cy3 (right). The error bars represent the accuracy of the fitting at the 95% confidence interval. **c** Statistical distribution of fluorescence intensity per fluorescence population density during SMLM imaging in QD-PAINT (red) and DNA-PAINT with Cy3 (black). The exposure times were 500 and 5000 ms for Cy3 and mQDs, respectively.

Next, we investigated mQD photoswitchability for QD-PAINT in cells (Fig. 2a). As transient hybridization and specificity can be tuned by changing the length and sequence of the DNA hybridization pair between the docking and imager strands, we reduced the length of the docking strands from 20 nucleotides to 8 nucleotides (Supplementary Table 1). The mQDs that were hardly dissociable in the 20-nucleotide hybridization between the docking and imager strands (Supplementary Video 2) became photoswitchable by transient hybridization with an on-time of ~17.8 ± 0.7 s (Fig. 2b and Supplementary Video 3). We compared the photoswitching and intensity profiles of QD-PAINT with those of DNA-PAINT with Cy3 (Supplementary Video 4). While the exposure time in QD-PAINT was 10-fold higher than that in DNA-PAINT with Cy3, the on-time was 8.9-fold higher in QD-PAINT than in DNA-PAINT with Cy3. This 8.9-fold difference in the on-time occurred because the hybridization sequence length in QD-PAINT was 1 nucleotide longer than that in DNA-PAINT with Cy3, and this result was consistent with previous studies that reported an 8- to 10-fold higher dissociation constant for a 1-nucleotide-longer hybridization sequence^32,33^. Interestingly, the mQDs exhibited a multimodal distribution of the fluorescence intensity profiles, with higher fluorescence intensity by ~10- to 80-fold than that in DNA-PAINT with Cy3, which had a unimodal distribution (Fig. 2c). The mQDs appeared to contain four different species that showed clear differential fluorescence intensities of up to ~9-fold between the mQD species with the highest and lowest fluorescence intensities. Considering the 8.9-fold higher on-time of QD-PAINT than that of DNA-PAINT with Cy3, the overall fluorescence intensity difference was ~8–9-fold, under the assumption that their on-times were similar, leading to a theoretical improvement in resolution by a factor of ~3.

### QD-PAINT improves the spatial resolution over that in DNA-PAINT with Cy3

**Fig. 3:**
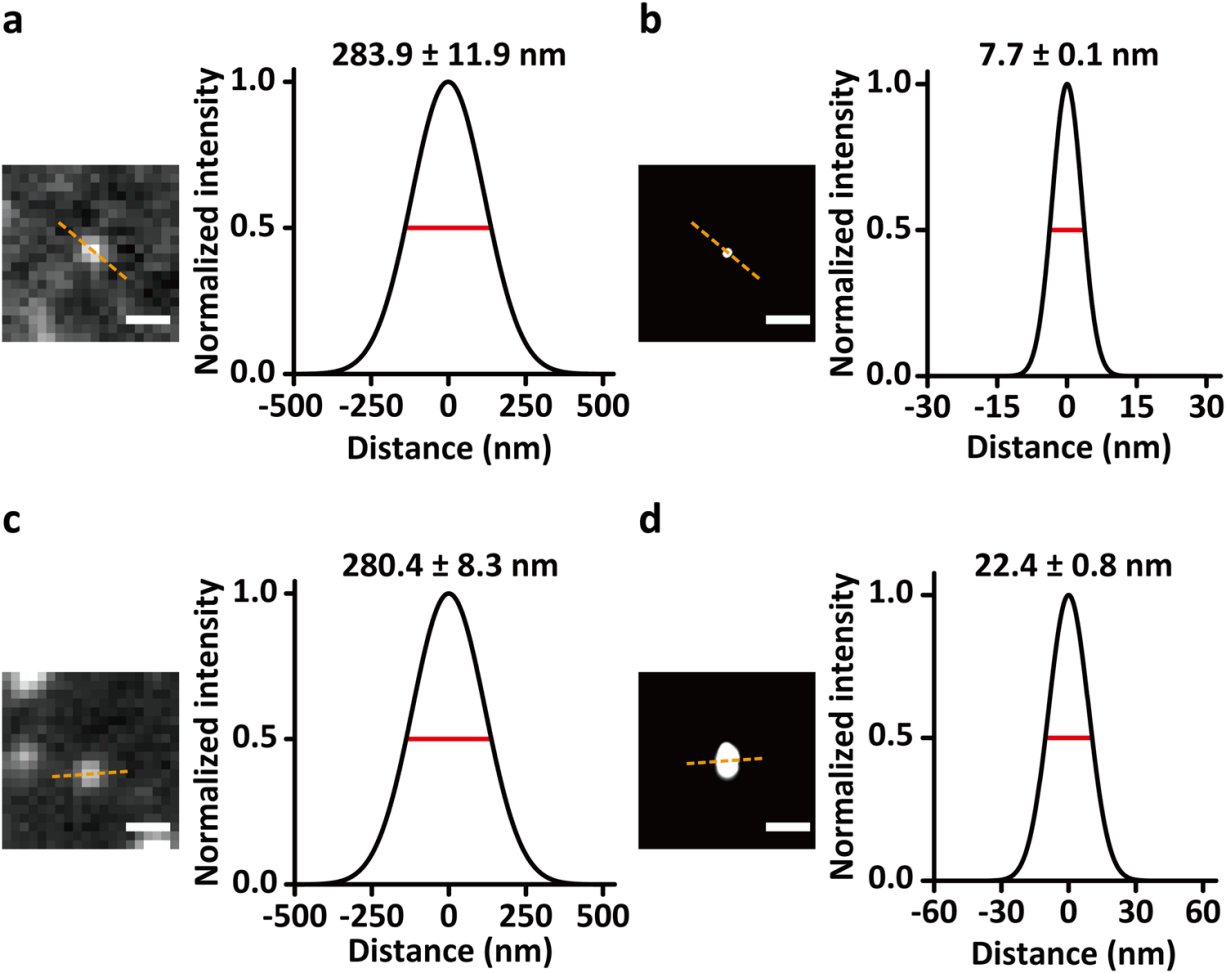
Improvement in spatial resolution with QD-PAINT over that in DNA-PAINT with Cy3. Representative single-molecule images of EGFR obtained using QD-PAINT before (**a** left) and after (**b** left) reconstruction and using DNA-PAINT with Cy3 before (**c** left) and after (**d** left) reconstruction. Scale bars, 500 nm (**a**, **c**) and 100 nm (**b**, **d**). Cross-sectional histograms of the single molecules of EGFR for measuring the full width at half maximum (FWHM) over the dashed lines using QD-PAINT before (**a** right) and after (**b** right) reconstruction and using DNA-PAINT with Cy3 before (**c** right) and after (**d** right) reconstruction.

We examined whether QD-PAINT can surpass the spatial resolution limit of DNA-PAINT with Cy3. We reconstructed EGFR images in QD-PAINT and DNA-PAINT with Cy3 (Supplementary Figs. 4, 5) and analyzed the single-molecule resolution of mQD- and Cy3-labeled EGFR (Fig. 3a–d). The full width at half maximum (FWHM) of the single molecules of mQDs and Cy3 in the TIRF images were similar, with values of 283.9 ± 11.9 nm and 280.4 ± 8.3 nm, respectively. After image reconstruction, the FWHM of the single molecules of mQDs and Cy3 in SMLM narrowed to 7.7 ± 0.1 nm and 22.4 ± 0.8 nm, respectively, showing an improvement in spatial resolution in QD-PAINT of 2.9-fold. One proper way to estimate the imaging localization precision is to distinguish two closely localized imaging points^34^. We estimated the imaging localization precision of QD-PAINT after reconstruction by measuring the distance between the maximum of each PSF of two closely localized SNAP-EGFR pairs. QD-PAINT was capable of resolving two SNAP-EGFR pairs separated by ~8 nm (Supplementary Fig. 6).

## Discussion

In this study, we introduce a promising next-generation SMLM method named QD-PAINT that increases the spatial resolution of conventional SMLM by utilizing the high brightness of QDs. Until now, sub-20 nm spatial resolution has been feasible in a study using DNA-PAINT in vitro by trace averaging, templated, and geometry-templated drift correction with origami as fiducial markers, with a photon count cutoff, achieving a resolution of ~5 nm^35^. QD-PAINT shows great promise for achieving sub-20 nm spatial resolution in vivo by fundamentally increasing the photon number that critically determines the spatial resolution. Our methodology overcame the major limitations of QDs in the utilization of SMLM due to their high nonspecificity, multivalency, and, most importantly, nonphotoswitchability by combining steric exclusion and DNA-PAINT methods^7,36^. After passivation with ptDNA-PEG6-casein and tuning of hybridization sequences, monovalent and specific QDs became photoswitchable via QD-PAINT and maintained superiority to Cy3 in fluorescence intensity, showing a theoretical improvement in resolution by a factor of ~3. QD-PAINT achieved improved spatial resolution with a narrower FWHM than that of Cy3 by 2.9-fold.

Although bare QDs are generally known to be substantially brighter than organic dyes, the improvement in the localization accuracy of QD-PAINT was limited to an ~3-fold increase derived from the ~9-fold higher fluorescence intensity than that of Cy3 (Figs. 2c and 3). As previously reported regarding the diminishing effect of thiol ligands on the fluorescence intensity of QDs^37,38^, this unexpectedly ‘reduced’ fluorescence intensity of mQDs is also probably due to the caging effect induced by the thiol ligands used in the preparation of monovalent QDs, including mPEG thiol, ptDNA, and carboxy PEG6 alkane thiol. However, as shown in Fig. 2c, the fluorescence intensity profile of mQDs showed a multimodal distribution, suggesting the inclusion of multiple mQD species with differential fluorescence intensities ranging from 10- to 100-fold higher than that of Cy3. The ‘reduced’ fluorescence intensity could be recovered by uncaging mQDs passivated with surface-bound thiol molecules upon laser excitation (Supplementary Video 5), as also observed in a previous report^39^. Therefore, the mQDs with a fluorescence intensity of 10^4^ in Fig. 3 may be responsible for the 9-fold higher intensity of mQDs than Cy3, and the mQDs with a fluorescence intensity of 10^5^, thus 100-fold higher intensity than Cy3, may lead to a 10-fold improvement in resolution. To utilize the brightest mQDs with further increased photon output for SMLM, sufficient pre-excitation with laser illumination could be employed. Although the proof-of-concept of QD-PAINT led to a limited resolution improvement of a 3-fold increase, the current QD-PAINT in its primary stage of technique development has not reached its full potential, and further optimization to maximize the photon output will lead to further improved spatial resolution. In addition, given that the DNA-PAINT system allows for constant renewal of QDs on the sample by exchange with reservoir QDs from solution, QDs can be detected multiple times at each spot, enabling accumulation of photons for much higher localization precision and further improvement of the resolution.

The major limitation of the current QD-PAINT is the long image acquisition time required for the whole structure of the imaging sample compared to conventional SMLM. In the current QD-PAINT, each QD is detected only once, but due to the high quantum yield of QDs, substantially more photons can still be collected, resulting in improved resolution. Conceptually, as QD-PAINT with an on-time of ~17.8 s detects ~100 molecules in each frame every 5 s, the total acquisition time for imaging a total of 1 × 10^6^ molecules on the plasma membrane is ~49 h. Although a long image acquisition time is required, as the initial forms of SMLM techniques such as PALM and DNA-PAINT also took several hours up to days^9^, we focused on presenting a general method to generate the blinking of bright monovalent QDs required for SMLM through DNA-PAINT and steric exclusion with ptDNA and demonstrated the proof-of-concept of our method. For optimization of QD-PAINT, the balance between the on-time and off-time, typically quantified as a duty cycle in SMLM, critically contributes to the spatiotemporal resolution of superresolution images. The on-time is crucially proportional to the length of DNA linkers for hybridization between targets and mQDs. However, the off-time is not significantly affected by the length of DNA linkers but mainly depends on the concentrations of docking strand-bearing target proteins and imager strand-bearing mQDs because the hybridization between short DNA oligomers is primarily diffusion-limited. As the concentration of docking strand-bearing target proteins varies in different samples, the concentration of imager strand-bearing mQDs should be adjusted sample-by-sample to optimize the off-time. For practical use of QD-PAINT, extensive optimizations, including the on-time and off-time, will be required in a further study. Upon optimization, the image acquisition time of QD-PAINT may be reduced down to a similar extent as in the speed-optimized DNA-PAINT methods using various approaches^40–42^, in which resolving 20-nm distances with sufficient sampling in 5 min is possible. The optimized imaging speed will allow the extremely photostable QDs to be detected multiple times at each spot, leading to the accumulation of photons for higher localization precision and further improvement of the resolution within an overall reasonably short image acquisition time.

The large hydrodynamic size of mQDs is not expected to affect their labeling efficiency in terms of density or fidelity because mQDs detach from the binding site, allowing binding of another mQD to the same spot. The large hydrodynamic size of the mQDs can instead be advantageous by sterically hindering the binding of second molecules at close positions during the time they bind to the target molecules. This is preferable in SMLM because it reduces the chance of simultaneous binding of two or more QDs within the same diffraction-limited region.

Another potential application of QD-PAINT is the imaging of live specimens with the resolution of dynamic target molecules at a higher resolution than in sptPALM, which typically utilizes photoactivatable or photoswitchable fluorescent proteins or organic dyes^43^. As QDs absorb many more photons than organic dyes at the same excitation photon flux and subsequently release a higher number of photons, a low laser power can be sufficient for detecting QD-labeled molecules, avoiding the phototoxic effects on live cells of high-power laser illumination^44^.

Ultraresolution QD-PAINT will enable the fluorescence imaging of organelles and molecular complexes at the nanoscale, a level that has not been properly observed due to the limited resolution of the current SMLM that functions by utilizing photoactivatable or photoswitchable fluorescent proteins or organic dyes^45^. From the perspective of basic biology, the composition and molecular architecture of protein complexes or dense protein networks will be revealed, while alterations may become critical markers of disease conditions in the health industry. By realizing the unseen, QD-PAINT may become a cornerstone, if not the vanguard, of ultraresolution SMLM.

## Supplementary information

Supplementary figures and a table

Supplementary Video. 1 Specific binding of mQD-imager strand to SNAP-EGFR in a live COS7 cell

Supplementary Video. 2 Permanent binding of mQD-imager strand to SNAP-EGFR in a fixed and permeabilized COS7 cell via 20-nucleotide hybridization

Supplementary Video. 3 QD-PAINT on a fixed and permeabilized COS7 cell

Supplementary Video. 4 DNA-PAINT using Cy3-imager strands on a fixed and permeabilized COS7 cell.

Supplementary Video. 5 Increasing fluorescence intensity of mQD during imaging
