# Supplementary material for: Improved resolution in single-molecule localization microscopy using QD-PAINT
Source: Exp Mol Med. 2021 Mar 2;53(3):384–92. doi: 10.1038/s12276-021-00572-4 (PMC8080769; doi:10.1038/s12276-021-00572-4)
Supplement: Supplementary file 1 — Supplementary figures and a table [file 12276_2021_572_MOESM1_ESM.docx]

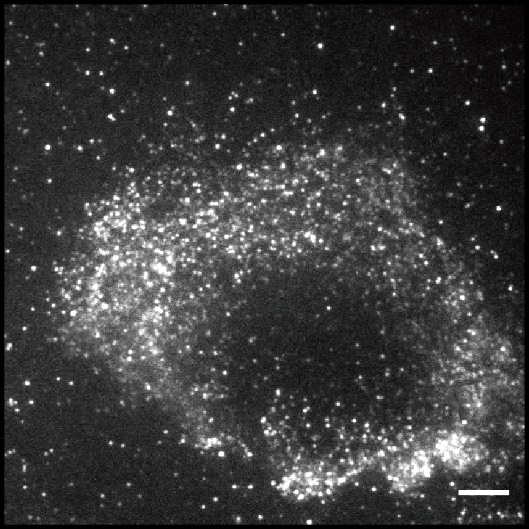


**Supplementary Fig. 1 Binding of the mQDs to SNAP-EGFR expressed in a fixed COS7 cell**

A representative TIRF image of the mQD-imager strands labeled on the BG docking strands covalently attached to SNAP-EGFR via 20 nucleotides, showing hindered binding to the central zone of a fixed COS7 cell. Scale bars, 5 μm.


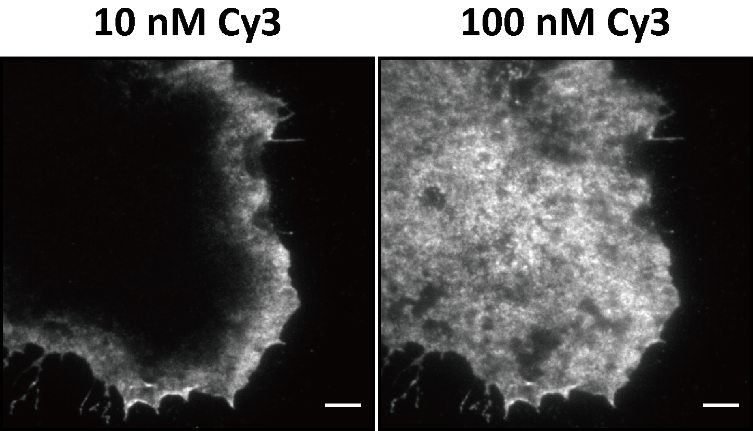


**Supplementary Fig. 2 Binding of the Cy3 imager strand to SNAP-EGFR expressed in a fixed COS7 cell**

Representative TIRF images of the Cy3-imager strands labeled on the BG-docking strands covalently attached to SNAP-EGFR, showing hindered binding to the central zone of a fixed COS7 cell. Scale bars, 5 μm.


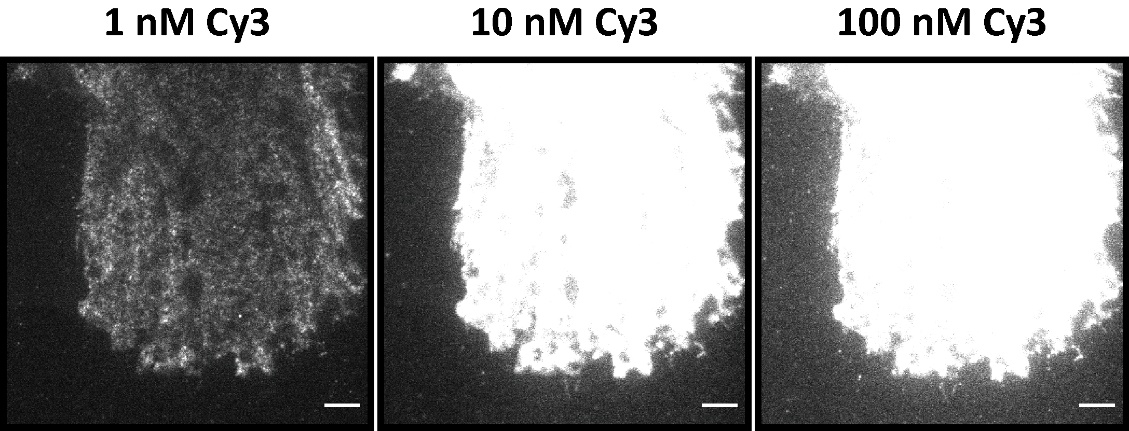


**Supplementary Fig. 3 Binding of the Cy3 imager strand to SNAP-EGFR expressed in a fixed-permeabilized COS7 cell**

Representative TIRF images of Cy3-imager strands labeled on BG-docking strands covalently attached to SNAP-EGFR, showing improved binding throughout a fixed and permeabilized COS7 cell. Scale bars, 5 μm.


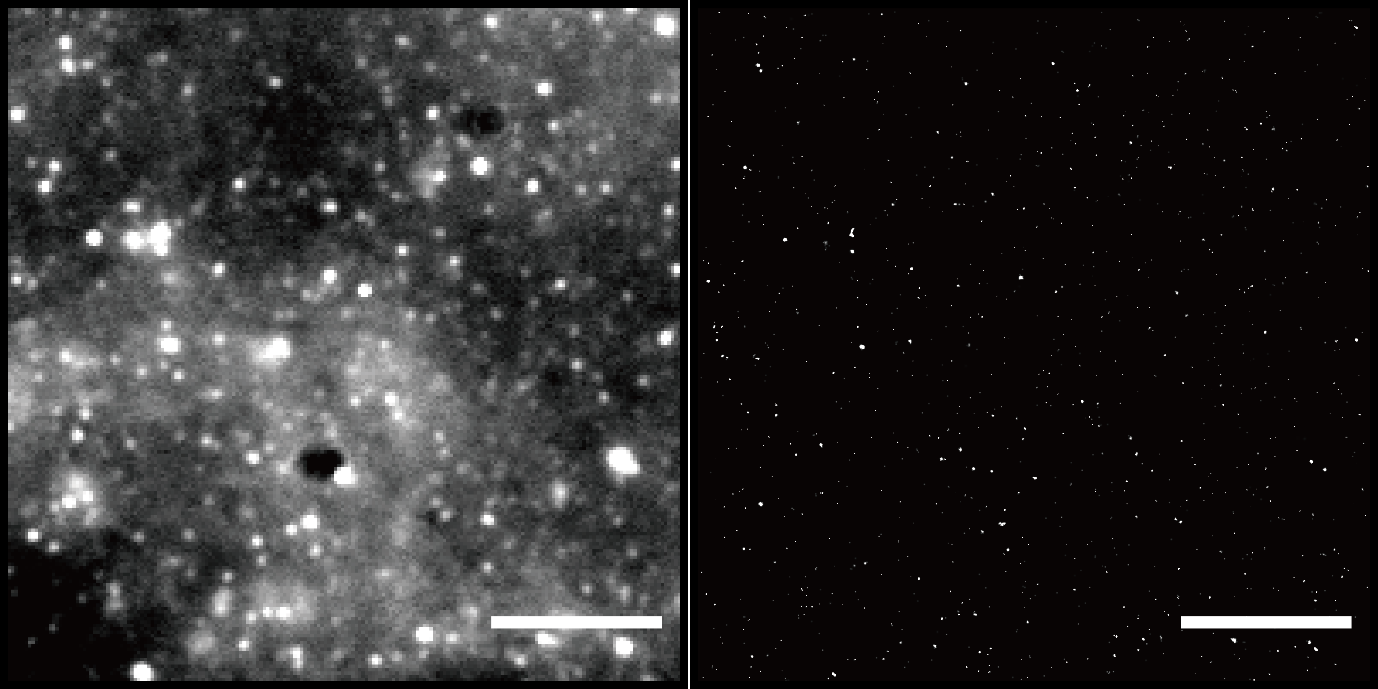


**Supplementary Fig. 4 TIRF and reconstructed images of QD-PAINT in a cell**

Representative TIRF (left) and reconstructed (right) images of QD-PAINT on EGFR in a COS7 cell. The data shown in Fig. 3a and 3b were generated from these data. Scale bars, 5 μm.


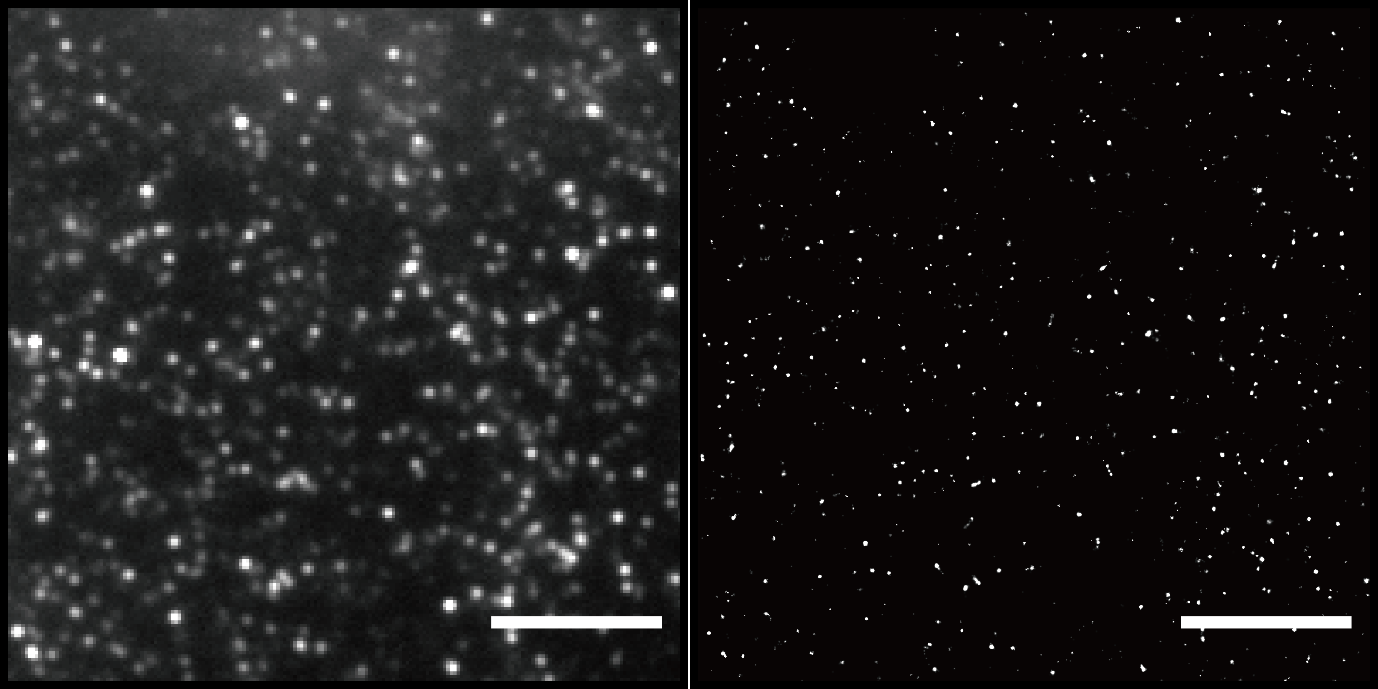


**Supplementary Fig. 5 TIRF and reconstructed images of DNA-PAINT with Cy3 in a cell**

Representative TIRF (left) and reconstructed (right) images of DNA-PAINT with Cy3 in EGFR in a COS7 cell. The data shown in Fig. 3a and 3b were generated by these data. Scale bars, 5 μm.


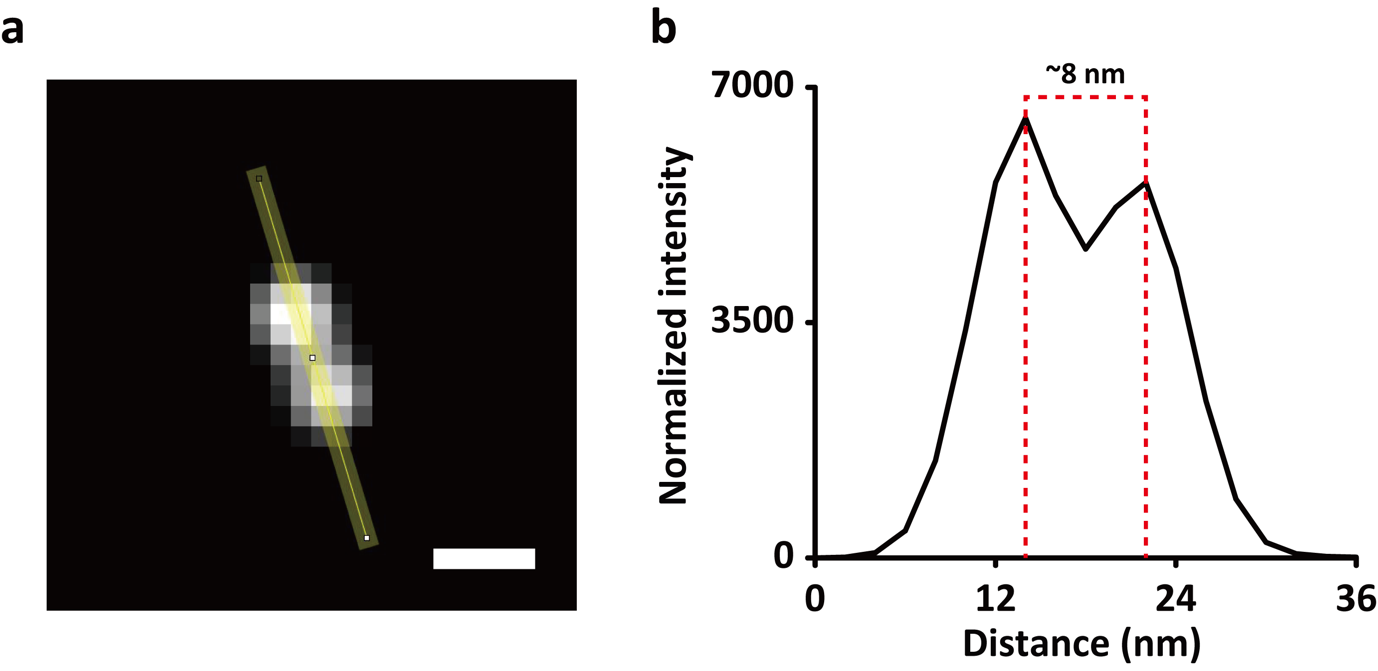


**Supplementary Fig. 6 The improved localization precision of QD-PAINT capable to resolve two SNAP-EGFR separated from each other by ~8 nm.**

**a** Representative reconstructed single-molecule image of two closely localized EGFRs using QD-PAINT. Scale bars, 10 nm. **b** Cross-sectional histogram of two single molecules of EGFR for measuring the distance between the maximum of each PSF of QDs on EGFR over the yellow line using QD-PAINT.

**Supplementary Table 1 Oligonucleotide sequences of docking and imager strands**

|  | **Oligo name** | **Sequence** |
| --- | --- | --- |
| **20-nt** | **Cy3 Imager strand** | /5Cy3Sp/ CTCTCTCTCTCTCTCTCTCTACTGACTGACTGACTGACTG |
|  | **QD Imager strand** | A*A*A*A*A*A*A*A*A*A*A*A*A*A*A*A*A*A*A*A*A*A*A*A*A*A*A*A*A*A*A*A*A*A*A*A*A*A*A*A*A*A*A*A*A*A*A*A*A*A*CTCTCTCTCTCTCTCTCTCTACTGACTGACTGACTGACTG |
|  | **Docking strand** | /5AmMC6/ CTCTCTCTCTCTCTCTCTCTCAGTCAGTCAGTCAGTCAGT |
| **QD-PAINT** | **Imager strand** | A*A*A*A*A*A*A*A*A*A*A*A*A*A*A*A*A*A*A*A*A*A*A*A*A*A*A*A*A*A*A*A*A*A*A*A*A*A*A*A*A*A*A*A*A*A*A*A*A*A*CTCTCTCTCTCTCTCTCTCTACTGACTGACTGACTGACTG |
|  | **Docking strand** | /5AmMC6/ CTCTCTCTCTCTCTCTCTCTCAGTCAGT |
| **DNA-PAINT with Cy3** | **Imager strand** | /5Cy3Sp/ CTCTCTCTCTCTCTCTCTCTCAGTCAGTCAGTCAGTCAGT |
|  | **Docking strand** | /5AmMC6/ CTCTCTCTCTCTCTCTCTCTCAGTCAG |
